# Obituary

**Published:** 1889-02

**Authors:** 


### OBITUARY.

Dr. J. J. P. Ostrader, a well-known dentist, and prominent and honored citizen of Hot Springs, Ark., died at the residence of his son-in-law, Dr. W. A Thompson, Nov. 21, 1888, at the ripe age of sixty-nine years. The son of a New York farmer of moderate circumstances, he acquired a thorough education by his own exertions, graduating from the Boston Medical College. He was engaged in mercantile and journalistic pursuits for a number of years, in Michigan and Illinois, before he settled down to the practice of dentistry. Having attained prominent position and moderate wealth, he retired from active business some four years ago.

